# The association between age- and sex-related differences in muscle strength and physical performance in Chinese older adults: a 5-year prospective cohort study

**DOI:** 10.1007/s40520-025-03250-x

**Published:** 2025-11-26

**Authors:** Xuemei Lu, Jinghua Xia, Yanzhen Hu, Min Qian, Ling Wang, Yi Yuan, Dan Zhao, Shuangshuang Wang, Qingqing Zang, Kaiping Liu, Junxin Hu, Xiaoguang Cheng, Giuseppe Guglielmi

**Affiliations:** 1 Department of Nursing, Beijing Jishuitan Hospital, Capital Medical University, Beijing, China; 2 Department of Radiology, Beijing Jishuitan Hospital, Capital Medical University, Beijing, China; 3 Department of Clinical and Experimental Medicine, Foggia University School of Medicine, Foggia, Italy

**Keywords:** Sarcopenia, Handgrip strength, Physical performance, Age, Sex

## Abstract

The global demographic shift towards an aging population highlights the increasing prevalence of age-related health concerns, such as sarcopenia, predominantly observed in older adults. This study aimed to evaluate the prevalence of muscle strength and physical performance in older Chinese adults and to elucidate the association between handgrip strength (HGS), the Timed ‘Up and Go’ (TUG) test, and various anthropometric indices (i.e., height, weight). Participants were enrolled from the China Action on Spine and Hip Status (CASH) study (NTC 01758770). Data were gathered through handgrip strength assessment, the TUG test, anthropometric measurements, and EQ-5D evaluations. All measurements were repeated after an interval of five years. An adjusted linear regression model was employed to examine the relationship between percentage changes in HGS and TUG and changes in anthropometric variables, including height, weight, body mass index (BMI), and EQ-5D in both sexes. The study included 125 participants, with 75 women (60%) averaging 67.6 years (SD 5.0) and 50 men (40%) averaging 69.0 years (SD 5.4). Over the five-year period of the study, HGS showed significant decrease, while TUG and EQ-5D showed significant increases when comparing the group aged below 70 years (*n* = 49) (HGS: -1.5 ± 21.0) with those aged 70 years and above (*n* = 26) (TUG: 33.8 ± 27.2; EQ-5D: 50.7 ± 27.7). In contrast, male participants did not exhibit any significant differences across all variables, including height, weight, BMI, HGS, TUG, and EQ-5D, when comparing the under-70 age group (*n* = 32) with those aged 70 or over (*n* = 18). There were no significant associations between the five-year percent changes in height, weight, BMI, and EQ-5D in both women and men, with the five-year percent changes in HGS (*p* > 0.05). The five-year percent changes in TUG were significantly correlated with changes in weight (β = 1.1, 95% CI: 0.1-2.0, *p* < 0.05) and BMI (β = 1.1, 95% CI: 0.1–1.9, *p* < 0.05) only in females. However, the percent changes in TUG for males did not significantly differ in relation to the five-year percent changes in height, weight, BMI, and EQ-5D in men and in height and EQ-5D in women (*p* > 0.05). In conclusion, our study demonstrated a significant decrease in HGS for individuals aged 70 and above. Notably, while the five-year percent changes in TUG in females were associated with weight and BMI, this may suggest a greater vulnerability of older women to poorer physical performance outcomes compared to men. Future research should focus on identifying key demographic groups and developing tailored intervention strategies to mitigate declines in physical function with age.

## Introduction

The global demographic landscape is undergoing a profound transformation, characterized by an accelerated aging population. This trend is not only reshaping societal dynamics but also significantly influencing global healthcare priorities. Among the most pressing concerns in this context is the increasing incidence and hospitalization rates among older adults, presenting a critical challenge in enhancing their functional independence and overall quality of life.

Sarcopenia, an increasingly recognized condition within the international medical community, represents a growing health concern for the aging population. Defined as a progressive, generalized skeletal muscle disorder marked by a reduction in muscle mass and physical function [1], sarcopenia has emerged as a significant barrier to physical health, garnering growing attention in both clinical practice and research. Prevalent among older adults, sarcopenia is now acknowledged as a global public health issue, necessitating the development of evidence-based definitions to facilitate the identification of clinically relevant outcomes [2].

Epidemiological studies report substantial variation in the prevalence of sarcopenia, which can be attributed to both subjective and objective factors influencing this demographic, as well as discrepancies in definitions [3]. Sarcopenia is commonly observed in older adults, with its prevalence increasing with age. A recent survey indicates that the global prevalence of sarcopenia ranges from 6% to 12%, with the prevalence among adults aged 65 years and older ranging from 14% to 33%. In the Chinese population, sarcopenia prevalence is reported to range from 8.9% to 38.8% [4]. Sarcopenia can lead to decreased physical activity, raising the risk of adverse outcomes such as falls and disability, while also placing a significant economic burden on patients and society, thereby negatively affecting the quality of life of older adults [5–7]. Overall, the prevalence of sarcopenia among individuals aged 60 and older is estimated at approximately 10%, regardless of sex [3].

The pathophysiology of sarcopenia involves a reduction in muscle volume and muscle fiber atrophy, influenced by various factors such as dietary habits, quality of life, physical health, and cultural background [8]. Age-related skeletal muscle deterioration imposes considerable limitations on physical functionality, often culminating in sarcopenia. Research highlights the importance of body movement coordination, excitation-contraction coupling, and overall bodily function in maintaining muscle strength, especially in the iliac muscles and the interconnected muscular, tendon, nerve, and skeletal systems in older adults [9]. Furthermore, the decline in physical activity capacity among older adults is closely linked to impairments in physical function and muscle strength [9]. Thus, sarcopenia is recognized as an age-related condition, intricately connected to lifestyle habits and the social environment. Its risk factors include advanced age, malnutrition due to poor diet, chronic underlying diseases, and reduced physical activity tolerance.

In this context, the present study, utilizing data from the China Action on Spine and Hip Status (CASH) study (NTC 01758770), seeks to (1) evaluate muscle strength and physical performance in older Chinese adults and (2) compare the basic characteristics of participants based on the HGS and TUG tests. Additionally, the study aims to explore the relationship between five-year changes in HGS and TUG results and other anthropometric measurements such as height and weight.

## Materials and methods

### Participants and setting

This study included a subcohort from the original participants to search for the musculoskeletal biomarkers in Beijing Jishuitan Hospital, Capital Medical University.

Participants performed quantitative CT (QCT) scans of the lumbar spine, hip, and mid-thigh at baseline (between March 2017 and June 2017) and 5-yr follow-up (between July 2022 and August 2022). Exclusion criteria were inability to complete HGS or TUG tests, missing CT scans or unacceptable image quality. The remaining subjects were 125 cohort members (72 females, 48 males) with acceptable image quality at both baseline and follow-up. The median follow-up time was 5 years. The study is reported by the STROBE guidelines [10].

### Ethics approval and consent to participate

All participants were recruited from the China Action on Spine and Hip Status (CASH) study (NTC 01758770), an study led by researchers at Beijing Jishuitan Hospital of Peking University, China [11]. The hospital ethics committee approved the study (approval number No. 201512-02). All participants’data were anonymized. All participants agreed to take part in the study by means of informed consent and also gave permission for their anonymized quotes to be used in research communication. All participants were free to leave the study whenever they wished and their welfare would not be affected by this waiver.

### Handgrip strength assessment

**Test method** Prior to the test, participants were interviewed to ascertain any upper extremity pain, use of prosthetic limbs, and history of upper extremity surgery. During the test, the non-dominant hand was allowed to hang naturally at the side without making contact with the body or exerting force. Participants were advised against swinging their arm, and they grasped the dynamometer with their dominant hand without allowing it to contact the body. The shoulder of the dominant hand was slightly abducted, maintaining the body’s longitudinal axis at a 45-degree angle. The test commenced with participants gripping the dynamometer firmly, exerting maximum effort, then releasing and recording the reading. Standardized instructions were provided to ensure consistency. After a one-minute rest, a second measurement was taken, replicating the first test’s steps, and the higher of the two readings was recorded. Prior to the measurement, the tester demonstrated the proper technique to the participant. Handgrip strength (HGS) of the dominant hand was measured using a Jamar dynamometer (Jamar^®^, Los Angeles, CA, USA), a validated tool for grip strength assessment [12]. The reproducibility of HGS measurements is well established [13]. Low handgrip strength was defined according to the Asian Working Group for Sarcopenia (AWGS) as less than 26 kg for men and less than 18 kg for women [14], while the European Working Group on Sarcopenia in Older People 2 (EWGSOP2) defined low muscle strength as below 27 kg for men and below 16 kg for women [15].

### Timed ‘Up and go’ test

The Timed ‘Up and Go’ (TUG) test was conducted using a meter rule, a stopwatch, a standard chair with a height of 45 cm, and a red cone marker. Prior to the test, participants were asked about any pain in various body parts (such as the neck, waist, and knees) and any orthopedic or surgical procedures they had undergone. In preparation, each participant sat upright on the chair with their back against the chair back, hands resting flat on their thighs, feet flat on the ground, and toes facing forward. Participants who normally used assistive devices, like crutches, were permitted to use these during the test. The timing started as soon as the subject’s back left the chair back, and they were instructed to walk around the marker placed 3 m away (in any direction of their choosing), return to the chair, and sit with their back against the chair back, which marked the end of the timing. If the participant’s back could not touch the chair back, the timing was calculated from the onset of body movement. Throughout the test, especially near the red marker cone, the safety of the subject was closely monitored. Participants were advised to walk at a pace that felt most comfortable and safe to them. The testing personnel used a standardized phrase, “Please go around the red marker and return to the chair as quickly as possible,” and demonstrated the test to the subject before commencing. The reliability of the TUG test has been demonstrated in older adults with an intraclass correlation (ICC) of 0.94 [16]. According to EWGSOP2 criteria, low physical performance is defined as a TUG test duration of 20 s or longer.

### Anthropometric parameters and EQ-5D measurement

Anthropometric parameters, including height, weight, hip circumference, and waist circumference, were measured prior to the handgrip strength assessment. These measurements were taken following standard procedures, with participants in light clothing and without shoes. Older participants were advised to avoid strenuous exercise or heavy physical activity before the test. For consistency, the same trained tester measured all participants. For the height measurement, participants were asked to remove their shoes and hats, stand barefoot on the height measurement device, and straighten their bodies to attain a proper posture, aligning the heel, sacrum, and shoulder blade. Weight was measured with participants in light clothing, standing still on the weighing scale without any side-to-side movement or hand support. Body mass index (BMI) was calculated as weight in kilograms divided by height in meters squared (kg/m²).

The Chinese version of the European Quality of Life Scale (EQ-5D) was utilized to assess quality of life [17]. This included the five dimensions of the EQ-5D health description system: mobility, self-care, daily living ability, pain and discomfort, anxiety and depression, and the visual analog scale (EQ-VAS) [18]. Investigators, trained uniformly, conducted the surveys item by item, ensuring responses were recorded accurately and completely. Each day, the survey was checked for completeness, gaps, logical errors, and other issues, which were promptly addressed. The time trade-off (TTO) method was employed to generate utility scores, where 1 indicates perfect health and 0 indicates death [19]. Previous studies have demonstrated that the EQ-5D instrument exhibits high repeatability (ICC >0.95) and internal consistency (Cronbach’s alpha >0.90) [20]. All measurements were repeated five years after an interval of five years.

### Statistical methods

The variables were normally distributed and are presented as the mean with standard deviation, and the mean differences are presented with a 95% confidence interval. P-values were derived from the Student’s T-test. One-way analysis of variance was performed to identify factors that, apart from the exposure variable, could influence the patient-reported outcome measures at 5 years. HGS and the TUG tests were analyzed and presented separately by age and sex. Multivariate linear regression analyses were utilized to investigate the association between changes in anthropometric measurements and changes in HGS and TUG. Unadjusted and adjusted odds ratios, along with 95% intervals were confidence. Statistical analysis was performed using IBM SPSS Statistics for Windows version 27.0 (IBM SPSS Inc., Chicago, IL, USA). Differences were considered significant at *p* < 0.05.

## Results

The study initially participants in 2017, with a subsequent follow-up after five years, ultimately including a total of 125 participants. At baseline, the cohort comprised 75 women (60.0%) with a mean age of 67.6 years (SD = 5.0) and 50 men (40.0%) with a mean age of 69.0 years (SD = 5.4). Significant changes were observed in several parameters over the five-year period. Among women, height (cm), HGS (in kg), the TUG (in seconds), and the EQ-5D showed statistically significant changes (P-value < 0.01). In the male participants, height (cm), weight (kg), HGS (kg), TUG (s), and EQ-5D also demonstrated significant changes (P-value < 0.01). Notably, there was no significant increase in the average BMI of either gender group at the five-year follow-up. Detailed comparisons of baseline characteristics and changes observed at the five-year follow-up are presented in Table 1.

**Table 1:** Participant characteristics

		Female (*n* = 75)		Male (*n* = 50)		Total (*n* = 125)
Age (y)	Baseline	67.6 ± 5.0		69.0 ± 5.4		68.1 ± 5.2
	5-year follow-up	72.7 ± 5.0		74.0 ± 5.5		73.2 ± 5.3
	P- value	< 0.01		< 0.01		< 0.01
Height (cm)	Baseline	158.7 ± 5.1		171.3 ± 5.6		163.7 ± 8.1
	5-year follow-up	158.0 ± 5.2		169.9 ± 5.6		162.8 ± 7.9
	P- value	< 0.01		< 0.01		< 0.01
Weight (kg)	Baseline	65.2 ± 8.6		73.3 ± 12.3		68.4 ± 11.0
	5-year follow-up	64.9 ± 9.0		71.5 ± 13.1		67.6 ± 11.3
	P- value	0.58		0.02		0.04
BMI (kg/m2)	Baseline	25.9 ± 3.0		24.9 ± 3.1		25.5 ± 3.1
	5-year follow-up	26.0 ± 3.2		24.6 ± 3.3		25.4 ± 3.3
	P- value	0.67		0.26		0.79
HGS (kg)	Baseline	21.7 ± 3.6		34.4 ± 5.6		26.8 ± 7.7
	5-year follow-up	20.2 ± 3.8		31.3 ± 7.2		24.6 ± 7.7
	P-value	< 0.01		< 0.01		< 0.01
TUG (s)	Baseline	8.0 ± 1.3		8.1 ± 1.1		8.0 ± 1.3
	5-year follow-up	11.4 ± 3.8		11.8 ± 4.5		11.6 ± 4.1
	P- value	< 0.01		< 0.01		< 0.01
EQ-5D	Baseline	0.6 ± 0.1		0.6 ± 0.1		0.6 ± 0.1
	5-year follow-up	0.9 ± 0.1		0.9 ± 0.2		0.9 ± 0.1
	P- value	< 0.01		< 0.01		< 0.01

BMI, body mass index; TUG, the Timed Up and Go test; HGS, handgrip strength; EQ-5D, EuroQol 5-dimension score

**Table 2:** Five-year percent changes by sex and age

	Height	Weight	BMI		HGS	TUG	EQ-5D
Gender							
Female (*n* = 75)	− 0.4 ± 1.2	− 0.3 ± 7.9	0.6 ± 8.3		− 5.2 ± 20.1	42.5 ± 35.2	57.4 ± 31.2
Male (*n* = 50)	− 0.8 ± 15	− 2.5 ± 6.9	− 1.0 ± 6.2		− 8.9 ± 16.2	46.5 ± 53.1	46.9 ± 40.4
P-value	0.15	0.12	0.26		0.26	0.61	0.11
Female							
Age (< 70) (*n* = 49)	− 0.4 ± 0.9	0.7 ± 8.2	1.5 ± 8.4		− 1.5 ± 21.0	33.8 ± 27.2	50.7 ± 27.7
Age (≥ 70) (*n* = 26)	− 0.5 ± 1.7	− 2.1 ± 7.1	− 1.1 ± 7.8		− 12.3 ± 16.2	58.7 ± 42.8	70.5 ± 34.0
P-value	0.82	0.15	0.20		0.03	0.01	< 0.01
Male							
Age (< 70) (*n* = 32)	− 0.7 ± 1.4	− 1.6 ± 6.1	− 0.4 ± 5.4		− 6.2 ± 16.0	41.6 ± 46.6	40.0 ± 42.0
Age (≥ 70) (*n* = 18)	− 1.0 ± 1.7	− 3.9 ± 8.0	− 2.0 ± 7.5		− 13.7 ± 15.9	55.3 ± 63.7	58.6 ± 35.5
P-value	0.47	0.27	0.37		0.12	0.39	0.12

BMI, body mass index; TUG, the Timed Up and Go test; HGS, handgrip strength; EQ-5D, EuroQol 5-dimension score

**Fig. 1:**
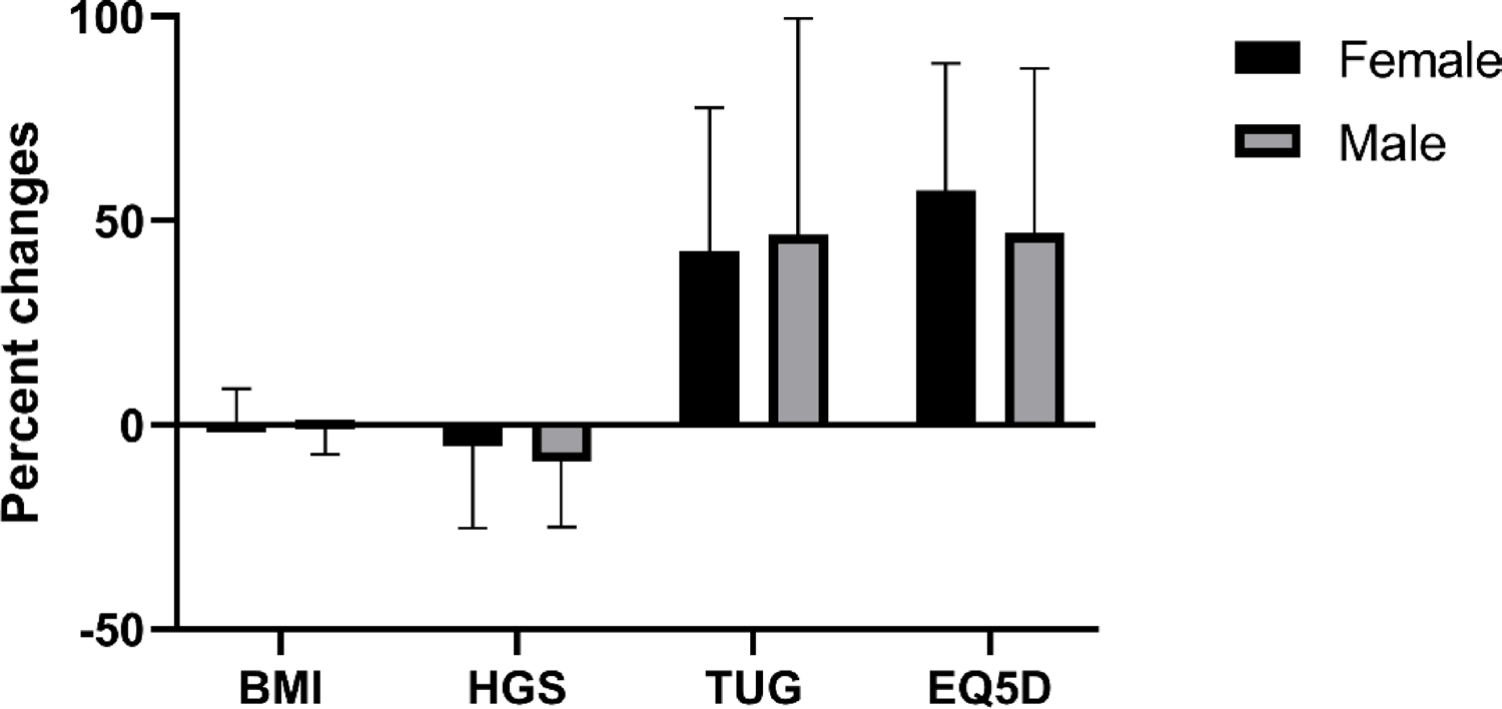
Box plot for five-year percent changes of BMI, HGS, TUG, EQ-5D in males and females

The analysis of five-year percent changes in various variables revealed no significant differences between sexes. However, in female participants, notable differences were observed when comparing age groups. Specifically, HGS showed significant decrease, and TUG and EQ-5D showed significant increase when comparing the group aged below 70 years (*n* = 49) (HGS: -1.5 ± 21.0) with those aged 70 years and above (*n* = 26) (TUG: 33.8 ± 27.2; EQ-5D: 50.7 ± 27.7). In contrast, male participants did not exhibit any significant differences across all variables, including height, weight, BMI, HGS, TUG, and EQ-5D, when comparing the under 70 age group (*n* = 32) with those aged 70 or over (*n* = 18), as detailed in Table 2. To visually represent these findings, a box plot illustrating the five-year percent changes in BMI, HGS, TUG, and EQ-5D in both sexes is presented in Fig. 1.

Multiple linear regression analysis indicated that there are no significant associations between the five-year percent changes in height, weight, BMI, and EQ-5D, both in women and men, with the five-year percent changes in HGS (*p* > 0.05). The five-year percent changes in TUG were significantly correlated with the changes in weight (β = 1.1, 95% CI: 0.1-2.0, *p* < 0.05) and BMI (β = 1.1, 95% CI: 0.1–1.9, *p* < 0.05) only in females. However, the percent changes in TUG of males did not significantly differ in relation to the five-year percent changes in height, weight, BMI, and EQ-5D in men and in height and EQ-5D in women (*p* > 0.05) (Table 3).

**Table 3:** Longitudinal association between changes in anthropometric measurements and changes in HGS and TUG, adjusted for baseline age

	Association with percent changes in HGS				Association with percent changes in TUG			
	Female (*n* = 75)		Male (*n* = 50)		Female (*n* = 75)		Male (*n* = 50)	
Changes in anthropometric measurements	β (95% CI)	P value	β (95% CI)	P value	β (95% CI)	P value	β (95% CI)	P value
Height	3.1(− 0.6, 6.8)	0.10	1.7(− 1.3, 4.6)	0.26	− 0.9(− 7.1, 5.2)	0.76	4.3(− 5.9, 14.6)	0.40
Weight	− 0.1(− 0.7, 0.5)	0.75	0.5(− 0.2, 1.1)	0.17	1.1(0.1, 2.0)	0.03	− 0.3(− 2.6, 2.0)	0.80
BMI	− 0.2(− 0.8, 0.4)	0.47	0.4(− 0.3, 1.1)	0.30	1.1(0.1, 1.9)	0.03	− 0.8(− 3.3, 1.7)	0.51
EQ-5D	0.1(− 0.2, 0.2)	0.91	0.1(− 0.1, 0.2)	0.13	− 0.1(− 0.3, 0.2)	0.57	− 0.1(− 0.4, 0.4)	0.90

TUG, the Timed Up and Go test; HGS, handgrip strength; EQ-5D, EuroQol 5-dimension score; CI, confidence interval.

## Discussion

Frailty, poor health and the loss of independence experienced with advanced aging is strongly associated with the age-related reductions in physical function and age-associated losses of muscle mass (sarcopenia) [21]. Epidemiological studies show a wide range of sarcopenia incidence, from 5% to 40% in older men and 7% to 70% in older women [22]. Reviews have indicated sarcopenia incidence rates in older adults (≥ 60 years) in various settings to be between 1.0% and 50.0% [23]. Studies by Wu et al. reported incidences in European [24], Taiwanese [25], and Turkish populations [26]. The variability in sarcopenia detection rates across populations is influenced by numerous factors, making direct comparisons challenging [27]. In this prospective cohort study, our findings revealed significant five-year percent changes in HGS, TUG, and EQ-5D in both male and female participants over a 5-year follow-up period. The research data showed that after 5 years, participants’ HGS values decreased, while TUG and EQ-5D values increased, both representing a trend of diminished muscle strength and physical performance after 5 years.

Males are stronger than females so the absolute rate of age-related decline in absolute strength and muscle mass is typically larger in males compared to females [28].Our findings align with this view, showing that the changes in HGS, TUG, and EQ-5D in females were more pronounced than in males, both in the < 70 years and ≥ 70 years age groups. Particularly in women aged ≥ 70 years, a more pronounced decline in muscle strength was observed. Sex-based analysis suggests a higher likelihood of muscle loss in women, potentially due to hormonal changes and related catabolism [29]. Our study’s findings align with this variability and highlight the increased incidence of sarcopenia with age [30].

The TUG test, involving simple movements including standing up, walking, turning, and sitting down, was utilized in this study. Studies on postural control indicate that TUG test times increase with age in both sexes, with women performing worse, possibly due to lower muscle mass and strength compared to men of the same age [31, 32]. The relationship between myogenic obesity and sarcopenia in older adults is a subject of debate. Obesity with sarcopenia might be linked to higher metabolic disorders and mortality risks [33]. The “obesity paradox,” where a higher BMI is associated with lower mortality, also complicates the narrative [34]. Sarcopenic obesity (SO), characterized by low muscle mass and a high BMI [35], presents unique challenges in older populations, as obesity may not correspond with increased muscle mass or function [36]. In our study, the BMI changes after 5 years were not significant, and regression analysis linked BMI with TUG changes in women, suggesting a potential protective role against sarcopenia.Notably, the 5-year percent changes in TUG were significantly correlated with the changes in weight and BMI in women, as shown in Table 1, where the change in weight (decrease) was not statistically significant. However, in Table 3, the regression analysis indicated a protective effect of weight and BMI changes. Additionally, as seen in Table 2, the weight change in women under 70 years was 0.7 ± 8.2, while in those aged ≥ 70 years, it was − 2.1 ± 7.1, highlighting a distinct trend among this older population, where maintaining or slightly increasing weight may have a positive effect on TUG.

There are some considerations to make when interpreting the results of our study. The focus on older adults in Beijing may not be generalizable to other adult populations, countries, or ethnic groups. Although we adjusted for confounders, including age, other factors, such as environmental and psychological influences, might affect the relationship between HGS, TUG, and demographic variables.

## Conclusions

In conclusion, our study demonstrated significant decreases in HGS, and increases in TUG and Eq. 5D over 5 years in terms of height, HGS, TUG, and Eq. 5D among older Chinese adults, especially in women aged ≥ 70. Notably, while the 5-year percent changes in TUG in females were associated with weight and BMI. This may suggest a potential greater vulnerability of older women to poorer physical performance outcomes compared to men. Future research should focus on identifying key demographic groups and developing tailored intervention strategies to mitigate declines in physical function with age. The broader impacts of these findings should be identified in further research.

## Data Availability

No datasets were generated or analysed during the current study.
